# Supplementary material for: Giant electrocaloric materials energy efficiency in highly ordered lead scandium tantalate
Source: Nat Commun. 2021 Jun 2;12:3298. doi: 10.1038/s41467-021-23354-y (PMC8172889; doi:10.1038/s41467-021-23354-y)
Supplement: Supplementary file 1 — Supplementary Information [file 41467_2021_23354_MOESM1_ESM.pdf]

Supplementary Information

Giant electrocaloric materials energy efficiency in highly ordered lead scandium tantalate

Youri Nouchokgwe[1,2*], Pierre Lheritier[1], C.-H. Hong[3], Alvar Torelló[1,2], Romain Faye[1], Wook Jo[3], C. R. H. Bahl[4] & Emmanuel Defay[1*]

[1]*Materials Research and Technology Department, Luxembourg Institute of Science and Technology, 41 rue du Brill, L-4422 Belvaux, Luxembourg*

[2]*University of Luxembourg, 2 avenue de l'Université, L-4365 Esch-sur-Alzette, Luxembourg*

[3]*School of Materials Science and Engineering, Ulsan National Institute of Science and Technology, Ulsan 44919, South Korea*

[4]*Department of Energy Conversion and Storage, Technical University of Denmark, Anker Engelunds Vej, 2800 Kgs. Lyngby, Denmark*

** Email: youri.nouchokgwe@list.lu ; emmanuel.defay@list.lu*

Contents:

Supplementary Note 1: XRD Scan of our Lead Scandium Tantalate (PST) sample 1
- Supplementary Figure 1

Supplementary Note 2: Zero-field DSC measurements
- Supplementary Figure 2

Supplementary Note 3: PST bulk ceramic under electric field
- Supplementary Figures 3, 4

Supplementary Note 4: Temperature range of our PST
- Supplementary Figure 5

Supplementary Note 5: EC effects in PST sample 2
- Supplementary Figure 6

Supplementary Note 6: Adiabatic temperature change of bulk ceramics PST
- Supplementary Figure 7

Supplementary Note 7: Gadolinium
- Supplementary Figure 8

Supplementary Note 8: Comparison PST versus Gd
- Supplementary Figure 9

Supplementary Note 9: IR Camera and electrical measurements
- Supplementary Figures 10, 11, 12, 13, 14
- Supplementary Table 1

Supplementary Note 10: PST driven supercritically at 40 kV cm^{-1}
- Supplementary Figure 15

Supplementary Note 11: Materials Efficiency of caloric materials
- Supplementary Figure 16

Supplementary Note 12: Polarisation -electric field loops
- Supplementary Figures 17, 18

Supplementary Note 13: Structure of PST bulk material
- Supplementary Figure 19

Supplementary Note 14: Potential Application of bulk PST
- Supplementary Figures 20, 21, 22

Supplementary Note 15: Electrocaloric Exchangeable heat

- Supplementary Figure 23

Supplementary Note 1: XRD Scan of our Lead Scandium Tantalate (PST) sample 1

The B-site cation order $\Omega \approx 0.98$ in powdered Lead Scandium Tantalate (PST) was calculated from the integrated intensities peaks (111) and (200) [1-2]. The relation below was used to obtain Ω with CoK_{α} radiation $(I_{111}/I_{200})_{theor,\Omega=1} = 1.4$ [1]. A density $\rho \approx 9071$ kg m^{-3} was obtained.

$$\frac{(I_{111}/I_{200})_{exp}}{(I_{111}/I_{200})_{theor,\Omega=1}} = \Omega^2$$

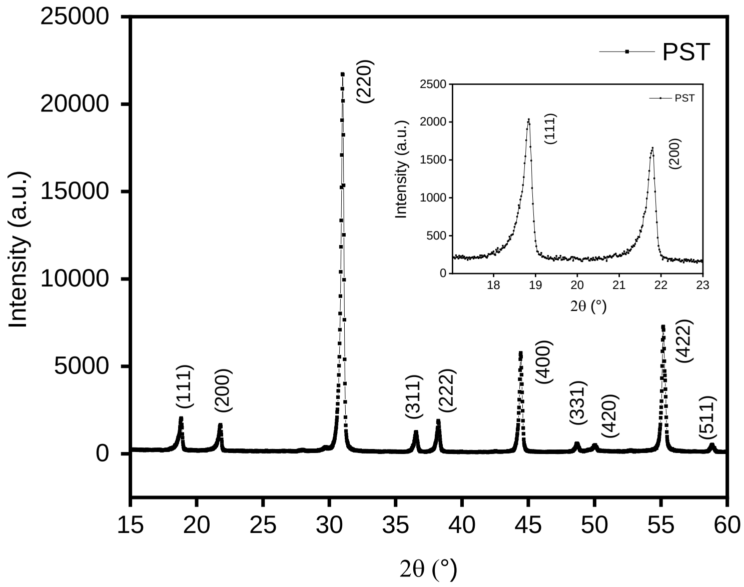

Supplementary Figure 1| **X-ray Diffraction of our PST powdered.**

Supplementary Note 2: Zero-field DSC measurements

The peaks of dQ/dT were integrated (trapezoid method) to get the latent heat Q_0 on cooling ($Q_{0,\mathrm{c}} = \int_{T_{c1}}^{T_{c2}} \frac{\mathrm{d}Q}{\mathrm{d}T} dT$) and heating ($Q_{0,\mathrm{h}} = \int_{T_{\mathrm{h1}}}^{T_{\mathrm{h2}}} \frac{dQ}{dT} dT$). T_{c1}, T_{c2} are respectively above and below the cooling peak. They are the extreme values for which heat flow dQ/dT is still naught before the latent heat peak starts appearing. T_{h1} and T_{h2} have been chosen similarly near the heating peak (cf Supplementary Fig. 2).

On cooling T_{c1}=300.523 K and T_{c2} = 288.019 K. On heating T_{h1} = 291.45 K and T_{h2} = 303.275 K.

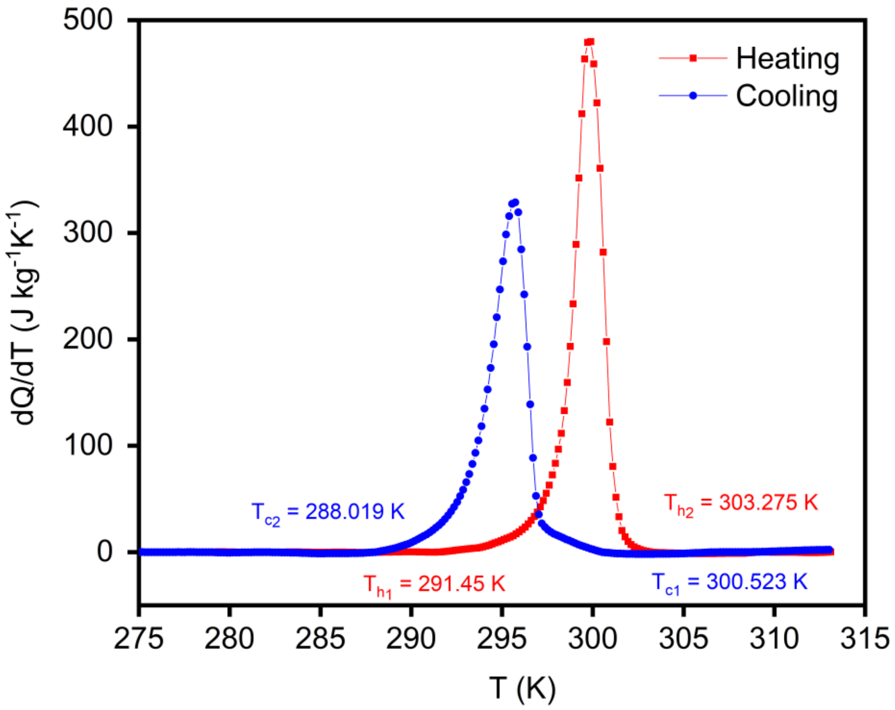

Supplementary Figure 2 | **Heat Flow measurements of PST on heating (red curve) and cooling (blue curve).**

Supplementary Note 3: PST bulk ceramic under electric field

From isofield measurements presented in Fig2a. we observe a linear shift of the transition temperature T_0 with electric field (Supplementary Fig. 3a.). Using the same data in Fig. 2a. we determine the latent heat at the transition (Supplementary Fig. 3b) as a function of electric field, thereby the entropy change at the transition temperature (Supplementary Fig. 3c). Here the measurements were carried out on a wired sample attached with silver paste (electrodes). The wires and electrodes induce an inferior thermal contact leading to a slight difference in the peak at zero field compared to Fig. 1b. However, the latent heat (integral under the peak) is the same in Fig 1b (1031 J kg^{-1}) and SI Fig 3d (1037 J kg^{-1}).

The measurements of C_p under electric field are quite challenging (Supplementary Fig. 3d.) because it is difficult to obtain a flat baseline. Indeed, the latter is affected by the connecting wires attached with silver paste to the sample. The results displayed in Supplementary Fig. 3d are representative of all the measurements performed under electric field. With the accuracy of our set-up, we could not deduce any variation of C_p baseline versus electric field. Consequently, C_p equals 300 J kg^{-1} K^{-1} +/- 20 J kg^{-1} K^{-1}, which is in line with literature [3]. The clear influence of electric field is C_p peak shifting towards higher temperature.

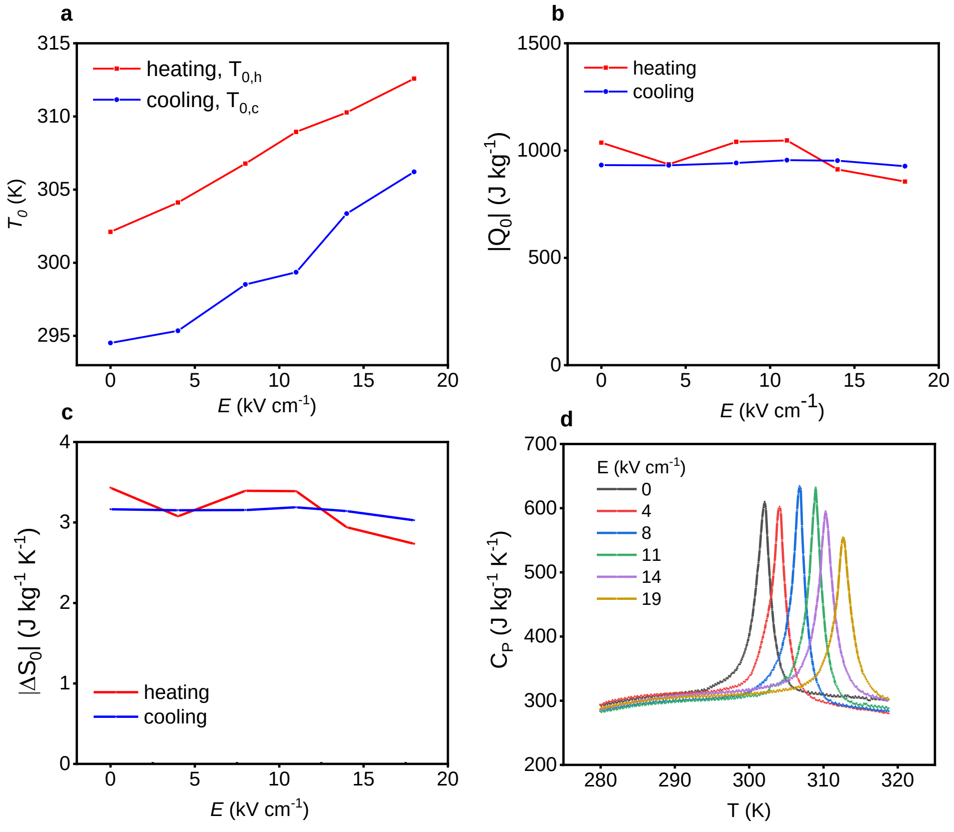

Supplementary Figure 3| **Temperature, latent heat, entropy change and specific heat deduced from isofield Differental Scanning Calorimetry (DSC) measurements on PST sample 1 a)** Transition temperature of PST versus electric field while heating $T_{0,h}$ (red curve) and cooling $T_{0,c}$ (blue curve) , **b)** latent heat Q_0 versus electric field, **c)** entropy change ΔS_0 versus electric field, **d)** Specific heat C_p measurements under electric field.

From specific heat measurements measured by differential scanning calorimetry (Supplementary Fig. 3d), we built the entropy curves (Supplementary Fig. 4) of our bulk ceramic PST referenced to 280 K using the relation below:

$$S'(T,E) - S(T = 280\text{K}, E) = \int_{280\text{ K}}^{T} \frac{\mathrm{d}Q(T',E)/\mathrm{d}T'}{T'} \mathrm{d}T'$$

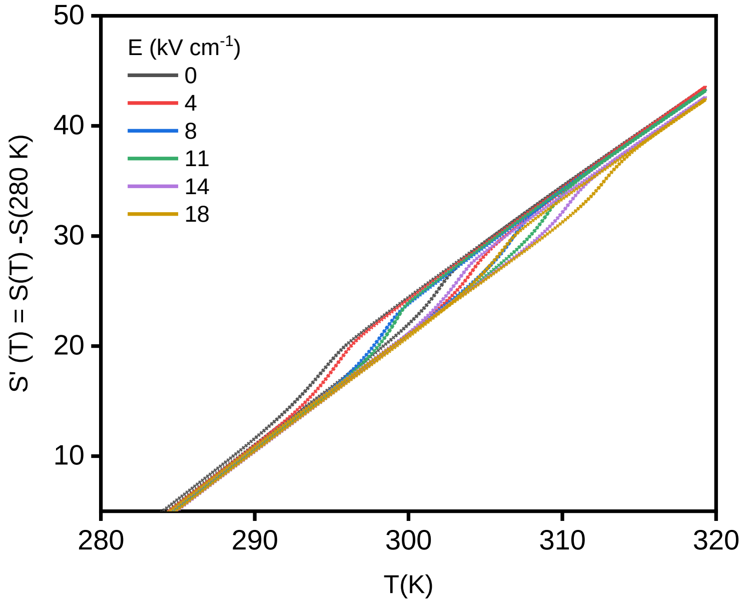

Supplementary Figure 4| **Entropy curves of bulk ceramic PST at five different electric fields upon cooling and heating.**

Supplementary Note 4: Temperature range of our PST

We observe a linear increase of the temperature range with the increasing field.

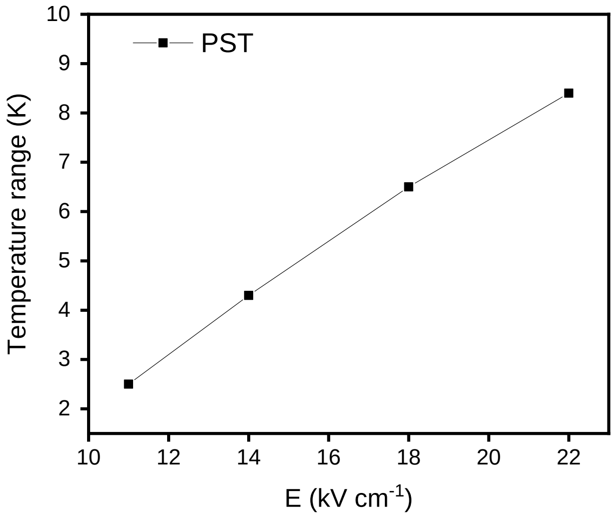

Supplementary Figure 5| **Temperature range of our PST.** The temperature range corresponds to the width of ΔT_{adiab} at 80% of its maximum value.

Supplementary Note 5: EC effects in PST sample 2

We measured direct ΔT_{adiab} on a second sample of PST using an IR camera. This sample has a B-site order of approximately 0.89. We obtain a reversible maximum ΔT_{adiab} of 2.4 K at 11 kV cm^{-1}.

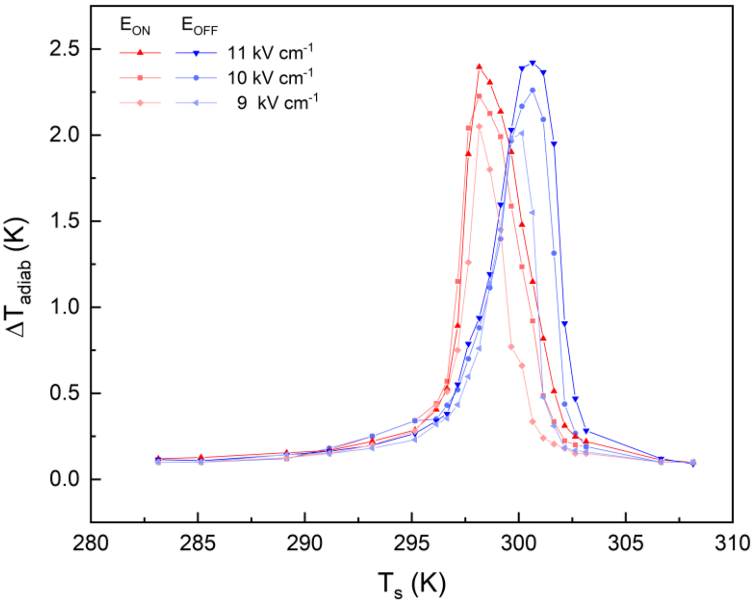

Supplementary Figure 6| **Adiabatic temperature change in PST sample 2.** This plot shows the ΔT_{adiab} of a less order PST (Ω=0.89) as a function of starting temperatures for three electric fields of 9, 10, 11 kV cm^{-1}.

Supplementary Note 6: Adiabatic temperature change of bulk ceramics PST

From data presented in Table 1, we plot the ΔT_{adiab} of bulk ceramics PST as a function of B-site cation order Ω. ΔT_{adiab} increases with Ω. Due to its high B-site order (0.98), our PST presents the highest ΔT_{adiab} of 3.7 K.

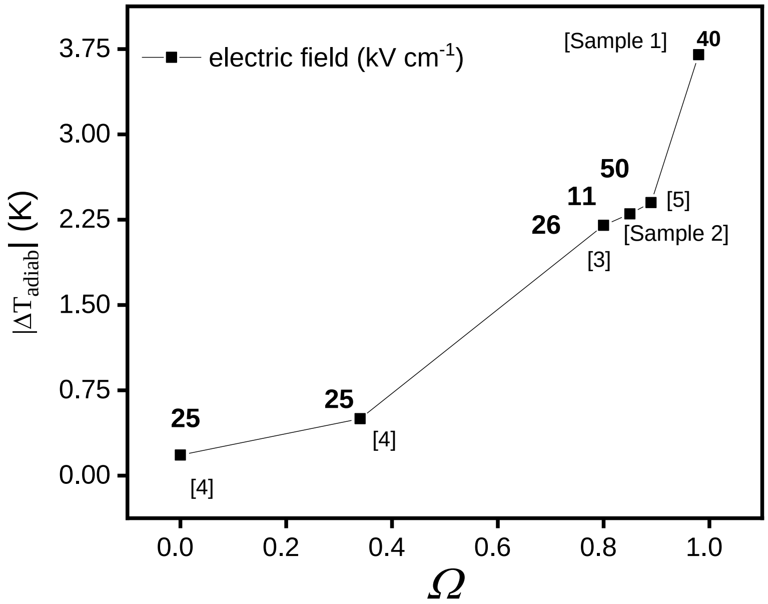

Supplementary Figure 7| **Adiabatic temperature change of bulk ceramics PST**. The bulk ceramics PST collected from literature [3-5] are compared to our PST samples (Sample 1, Ω=0.98 and Sample 2, Ω=0.89). ΔT_{adiab} of sample 1 data is shown in Supplementary Note 9 and ΔT_{adiab} of sample 2 are presented in Supplementary Note 5. The number displayed in bold closed to each symbol is the field applied in kV cm^{-1}. In brackets the references.

Supplementary Note 7: Gadolinium

Data of heat capacity C_p, adiabatic temperature change, and magnetization as a function of internal magnetic field were collected from Bjork et al. [6]. C_p measurements were carried out using a differential scanning calorimeter (DSC) with the magnetic field provided by a concentric Halbach cylinder. An average C_p value of 300 J kg^{-1} K^{-1} independent of the field applied was measured. ΔT_{adiab} measurements were done using a home-made instrument at DTU Energy, based on a pneumatic piston with the magnetic field provided by a concentric Halbach cylinder. The magnetization data was obtained using a LakeShore 7407 vibrating sample magnetometer. *Q, W*$_m$ and η_{mat} are presented in Supplementary Figs. 8c, 8e, and 8f respectively. We computed heat *Q* from Supplementary Figs. 8a and 8b, mechanical work W_m from Supplementary Fig. 8d and finally the materials efficiency η_{mat} in Gadolinium from Supplementary Figs. 8c and 8e.

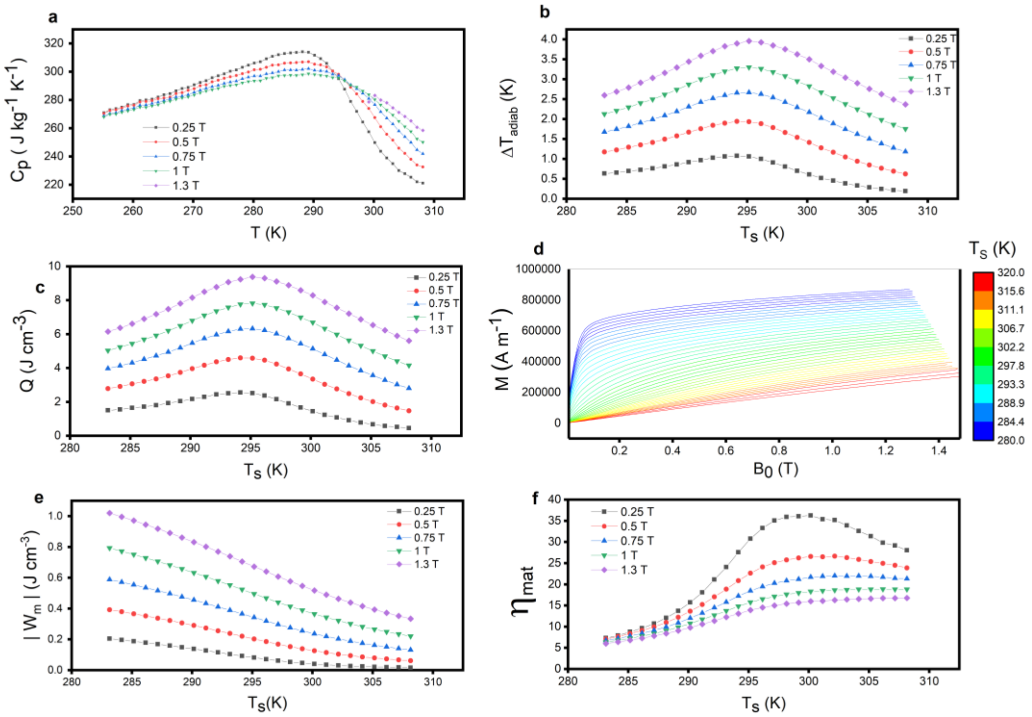

Supplementary Figure 8| **Materials Efficiency of Gd**. For five different internal applied fields B_0=$\mu_0 H$ (0.25, 0.5, 0.75, 1, 1.3 T) we show the heat capacity of Gd **a)**, the adiabatic temperature change **b)** and the magnetization at different temperatures **d)**. Computed *Q* **c)** and W_m **e)** used to obtained η_{mat} **f)** of Gd at different internal magnetic field B_0.

Supplementary Note 8: Comparison PST versus Gd

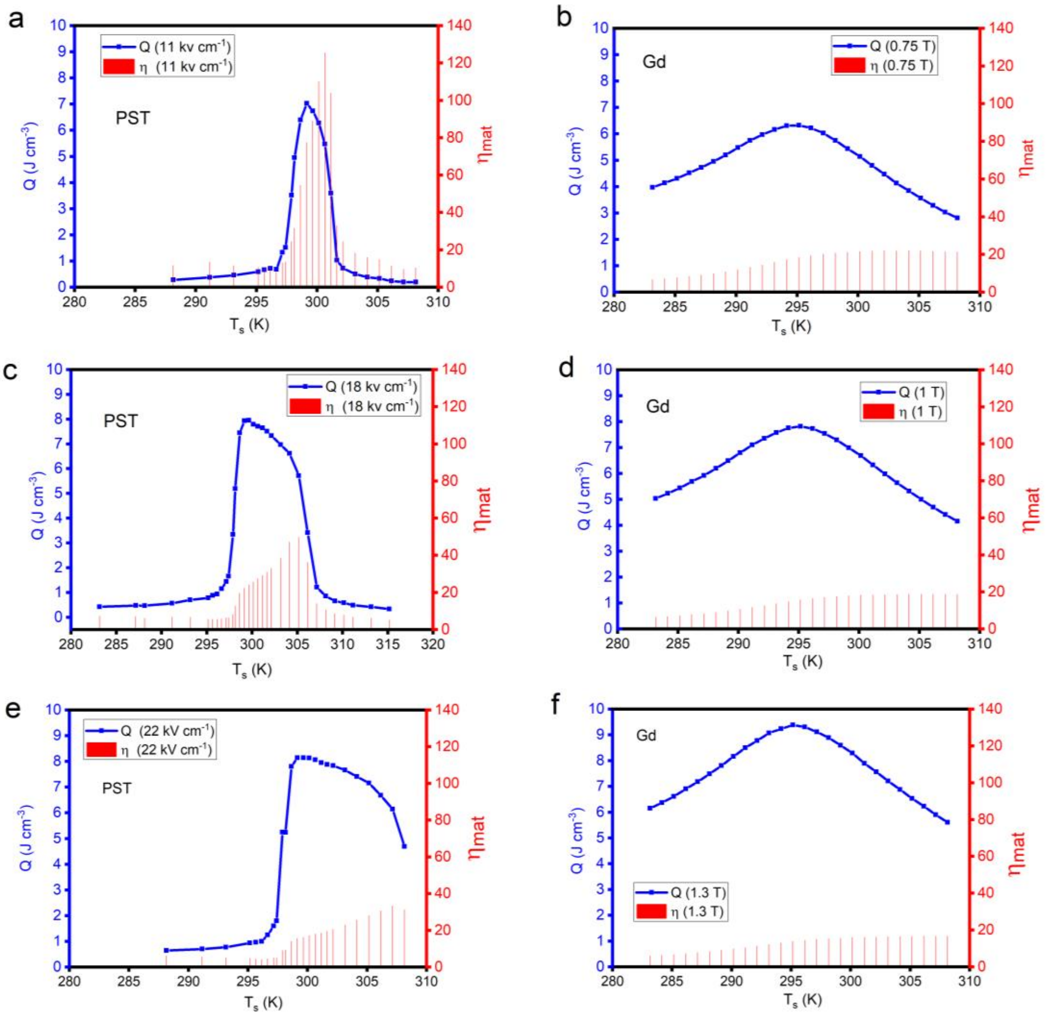

Supplementary Figure 9| **Comparison of materials efficiency in PST and Gd**. Each plot describes the heat exchanged *Q* per cm^{-3} (blue axis) and materials efficiency (red axis) of PST and Gd as function of starting temperature (black axis) for a given field. **a)** PST at 11 kV cm^{-1} **b)** Gd at 0.75 T **c)** PST at 18 kV cm^{-1} **d)** Gd at 1 T **e)** PST at 22 kV cm^{-1} **f)** Gd at 1.3 T.

Supplementary Note 9: IR Camera and electrical measurements

Here we present the measurements carried out to obtain Fig.3a. Adiabatic temperature change ΔT_{adiab} measurements were done at the four electric fields (11, 14, 18, 22 kV cm^{1}) from starting temperature T_s of 283 K to 323 K.

Measurements were taken every 0.5 K at the transition and 1 K out of the transition. For each measurement (points in Fig.3a) the material was first cool in the absence of electric field to 283 K (FE phase) and then heated to desired temperature where three electric field cycles were applied.

Supplementary Fig. 10a shows the measurements at the chosen T_s of 299 K, the temperature change of the material and three electric fields (voltage/thickness) cycles applied. An electric field cycle is made of 5 steps (numbers in blue in Supplementary Fig. 10a). In first step, the material is at given T_s (299 K) with no electric field applied. Subsequently, in step 2, an electric field is applied adiabatically (time of application of the field smaller than the characteristic time exchange with the surroundings) leading to a positive jump of the temperature of the material. The ΔT_{adiab} on field corresponds to the difference in temperature between the peak in step 2 and T_s. In step 3, the field is kept on and the material relaxes by exchanging with the surroundings. The material decreases to the initial T_s. This is a proof that the measurements are carried out without Joule heating. Afterwards, when the material is back to its initial $T_{s,}$ adiabatically the electric is removed to 0 kV cm^{-1} thereby a negative temperature jump is observed. The ΔT_{adiab} off field is the difference in temperature between the peak in step 4 and the T_s. Finally, the material relaxes and get back to its initial T_s. These five steps are repeated three times as presented in Supplementary Fig. 10a. At T_s and electric fields studied, we do not observe a decrease in the ΔT_{adiab} after several cycles (Supplementary Fig. 11); thereby showing the reproducibility of our measurements. The first, second and third peak of ΔT_{adiab} at the four electric fields (11, 14, 18, 22 kV cm^{1}) are presented Supplementary Fig. 11 An average of ΔT_{adiab} of the three cycles was done to obtain ΔT_{adiab} presented in Fig.3a.

Simultaneously to the ΔT_{adiab} measurements done by IR camera, we collected the charge of the PST. In Supplementary Fig. 10b, we present the charge of the bulk ceramic PST from 0 V to 1100 V (22 kV/cm) at a constant current of 0.2 mA. This charge corresponds to the step 2 in Supplementary Fig. 10a. The adiabaticity of the measurement is control by the current. The capacitor is charge in 0.12 s at constant current 0.2 mA. As one can observe in Supplementary Fig. 10b when the capacitor reaches its maximum voltage (1100 V) the current drops to 0 mA.

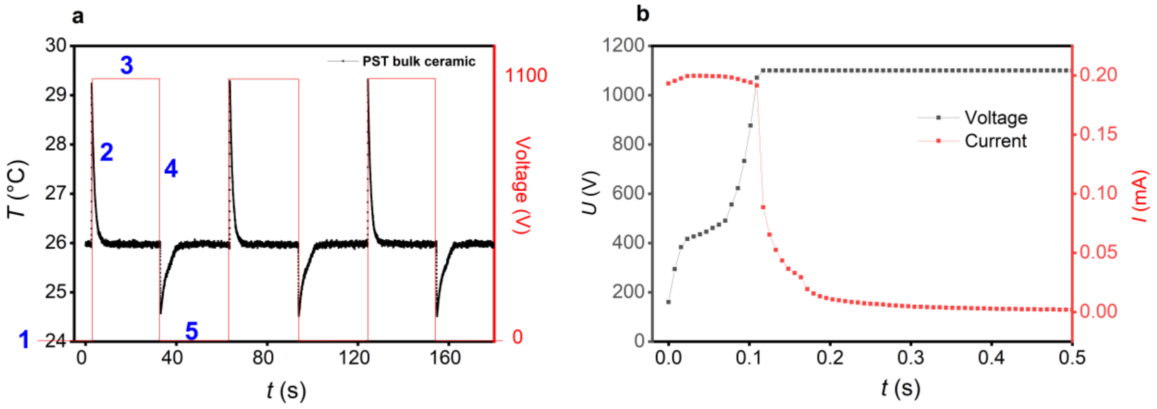

Supplementary Figure 10| **EC measurements description a)** Adiabatic measurements of PST bulk ceramic at 299 K. The black curve is the temperature of the material in function of time and the red curve represents the voltage in function of time applied to get the change in temperature. The numbers in blue described the different steps of an electric field cycle. **b)** Here we described the

charge of the capacitor (step 2 of the Supplementary Fig. 10a). The black curve shows how the voltage is charge to the desired maximum voltage. The red curve represents the change of current as a function of time. These data are collected simultaneously with the adiabatic temperature change data.

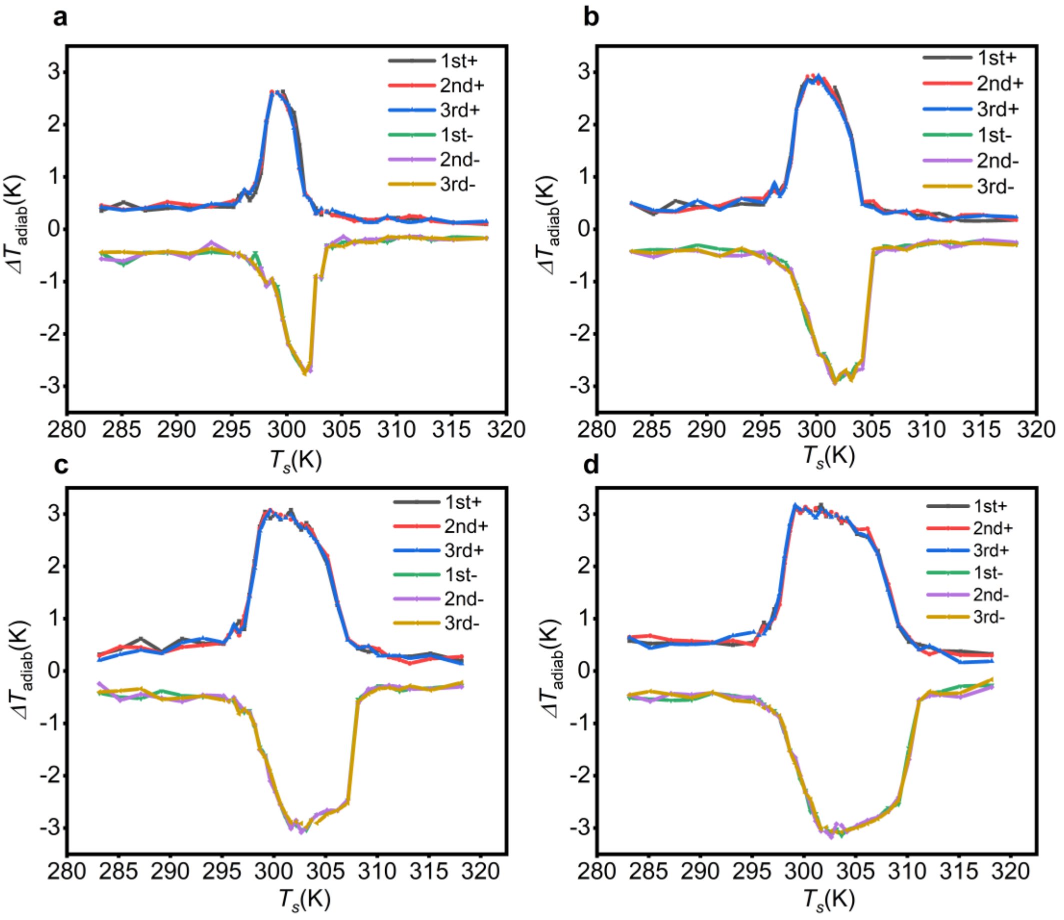

Supplementary Figure 11 | **Reproducibility of EC measurements** This figure shows the ΔT_{adiab} measured after of the first, second and third cycle in function of starting temperature T_s at 11 kV cm^{-1} **a)** 14 kV cm^{-1} **b)** 18 kV cm^{-1} **c)** 22 kV cm^{-1} **d).** In the legend, 1st+, 2nd+,3rd+ are the ΔT_{adiab} on-field of respectively the first, second and third cycle and, 1st-, 2nd-,3rd- are the ΔT_{adiab} off-field of respectively the first, second and third cycle.

Adiabatic conditions

The adiabaticity of the measurements is controlled by the current applied in our PST capacitor. The charging time of the sample must be much faster than the characteristic time of thermal relaxation to guarantee adiabaticity. As presented in Supplementary Fig. 12, for current lower than 0.2 mA, the thermal conditions are non-adiabatic because ΔT_{adiab} is lower than the asymptotic value of ΔT_{adiab}. When current ≥ 0.2 mA, ΔT_{adiab} stays constant and maximum, meaning that the expected adiabatic conditions are fulfilled. At 0.2 mA, the application/removal time of the electric field takes 0.12 s, which is about 30 times faster than the thermal relaxation time experienced by PST in our experiments (see Supplementary Fig. 10a).

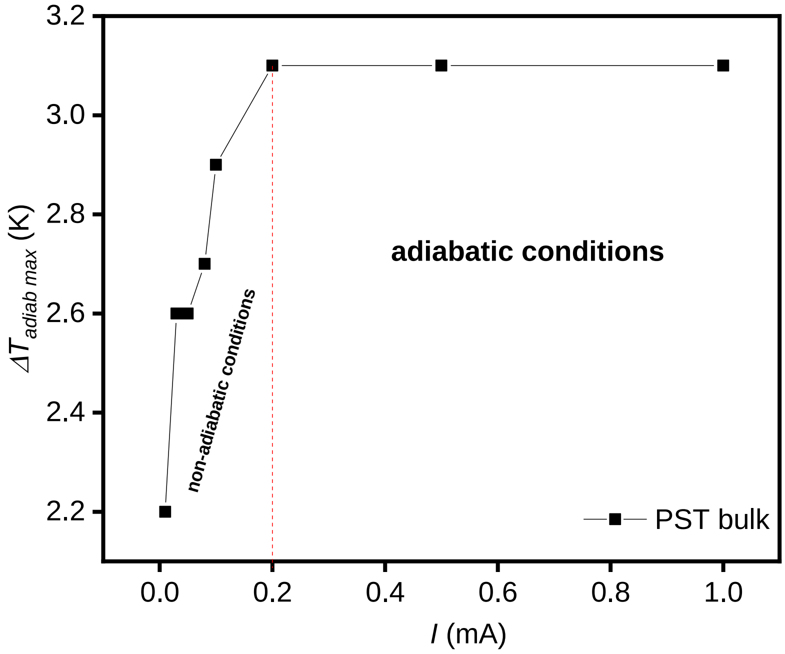

Supplementary Figure 12 | **Adiabatic conditions.** ΔT_{adiab} versus applied current. The measurements were done on PST sample 1 bulk at 22 kV cm^{-1}.

Asymmetry in adiabatic measurements

One can see in Fig. 3a and in Supplementary Fig. 11 that the adiabatic temperature changes at field on ($\Delta T_{adiab,on}$) and at field off ($\Delta T_{adiab,off}$) do not peak at the same starting temperature T_s.This is due to the reversibility of the electrocaloric effect because the temperature difference between the peaks is equal to the adiabatic temperature change (see Supplementary Fig. 13). This mechanism is explained in [7], in which they show that if a process is reversible, the relationship below must be verified: $\Delta T_{adiab,on}(T_s, E) = -\Delta T_{adiab,off}(T_s + \Delta T_{adiab,on}(T_s, E), E)$, with E the applied electric field. This is exactly what we observed in bulk PST.

The fact that ΔT_{adiab} field on is different in magnitude from ΔT_{adiab} field off is intrinsic to the material and due to the fact that the zero field and finite field entropy curves (Supplementary Fig. 4) get closer at low and high temperatures [8]. In the case of bulk PST, the difference in magnitude is large because of the very sharp first order transition. This asymmetry in the adiabatic temperature was also observed in PST MLCs [8-9] where the difference in magnitude is small due to the broad transition of PST MLCs. Asymmetric in ΔT_{adiab} was also reported in less ordered bulk ceramic PST [10] and in a 1st order transition magnetocaloric material (Fe-Rh) [11].

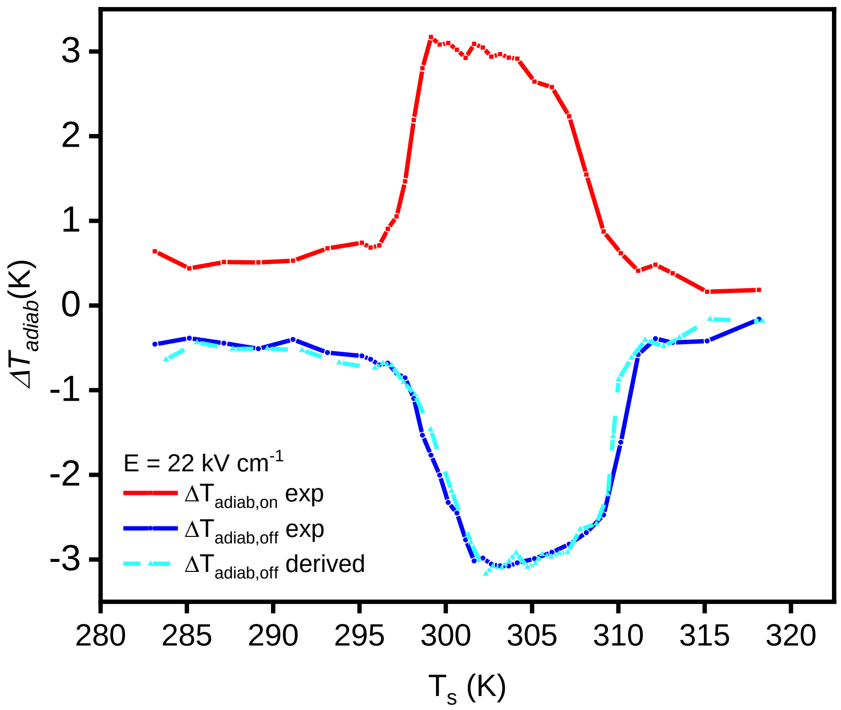

Supplementary Figure 13 | **Reversibility of EC measurements.** Here we show ΔT_{adiab} of PST bulk at 22 kV cm^{-1} versus starting temperature T_S. Red curves and dark blue curves are respectively ΔT_{adiab} due to the EC effect when the field is on and off and measured experimentally with an IR camera. The cyan dash curve is the ΔT_{adiab} due to EC effect when the field is off and derived from the equation above. $\Delta T_{adiab,off}$ exp and $\Delta T_{adiab,off}$ derived coincide well and thereby validate the reversibility of our EC measurements.

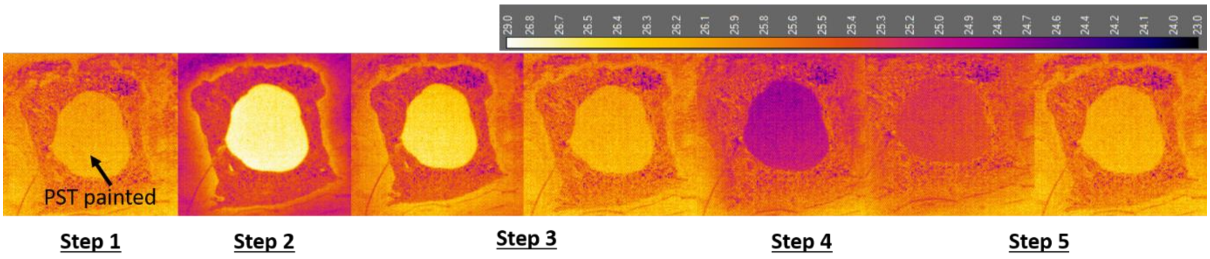

Supplementary Figure 14| **IR images scans of bulk PST sample 1 during a standard EC characterization sequence.** In step 1 the material is at starting temperature T_s of 299 K, in step 2, 1100 V are applied and the materials temperature increases. Subsequently, in the two following images the material thermalizes and goes back to T_s (no Joule heating). In step 4, the materials temperature decreases as voltage is removed. Finally, in Step 5 the material thermalizes back to T_s

Supplementary Table 1| **Voltage – Electric field**. Here we give the real voltage applied in the bulk ceramic PST sample 1 (0.5 mm-thick).

Voltage (V)	Electric Field (kV cm^{-1})
550	11
700	14
900	18
1100	22

Supplementary Note 10: PST driven supercritically at 40 kV cm^{-1}

On another sample from the same batch we applied field bigger than 22 kV cm^{-1}. The maximum field we could apply was 40 kV cm^{-1}. We measure around the transition temperature a maximum adiabatic temperature change of 3.7 K.

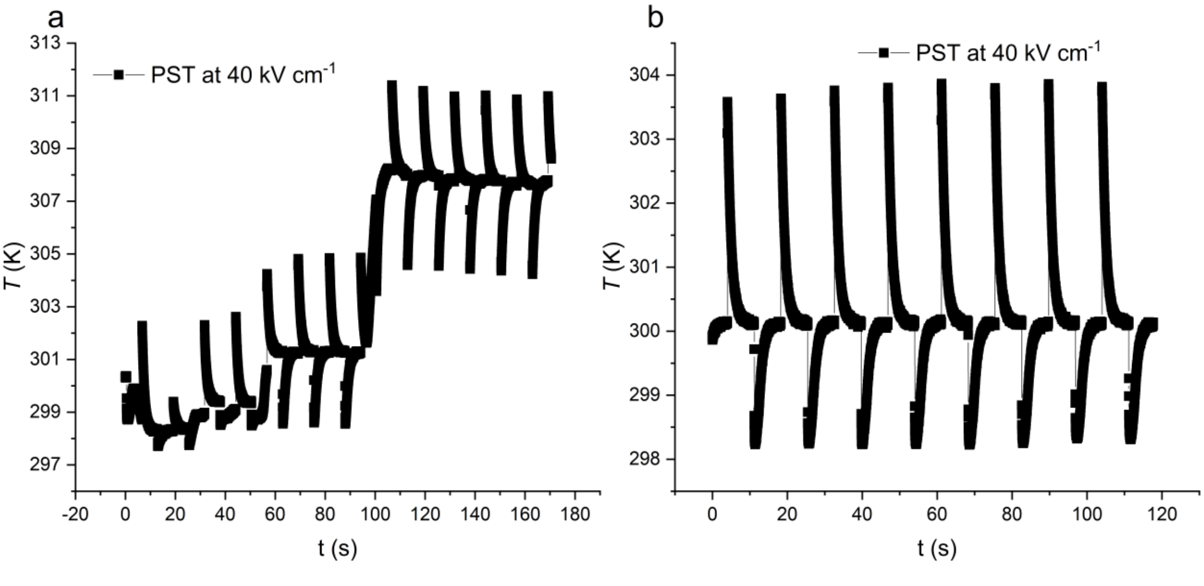

Supplementary Figure 15 | **PST driven at 40 kV cm^{-1}.** An electric field of 40 kV cm^{-1} was applied at temperature of 300 K, 301 K and 308 K.

Supplementary Note 11: Materials Efficiency of caloric materials

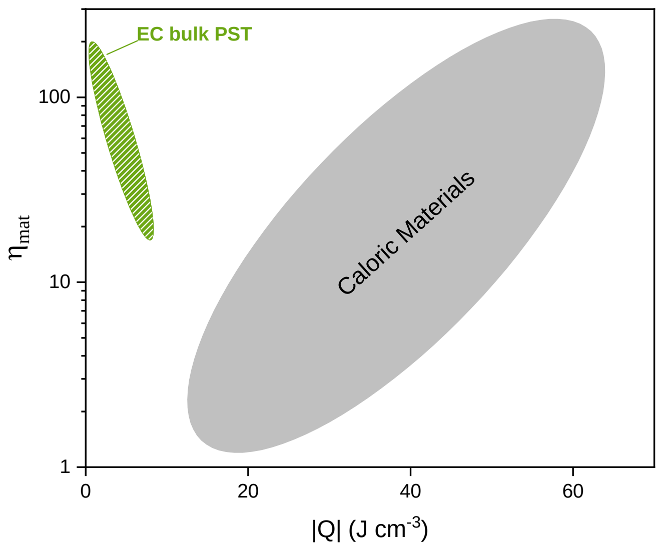

Supplementary Figure 16| **Comparison of materials efficiency of caloric materials.** This energy efficiency map of caloric materials as a function of the heat Q is adapted from Crossley et al. [12]. The caloric materials include magnetocaloric (MC) under permanent magnet of 2T, MC under solenoid, elastocaloric (eC) materials, barocaloric (BC), EC (electrically driven) and EC materials using energy recovery. In this map we added the materials efficiency of PST bulk. PST is shown here as being as efficient as the best caloric materials which are magnetocaloric (MC) under permanent magnet 2 T, barocaloric (BC) and EC materials using energy recovery.

Supplementary Note 12: Polarisation -electric field loops

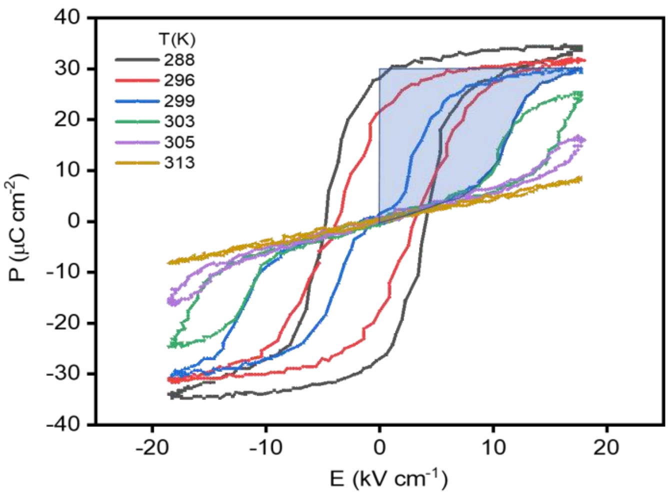

Supplementary Figure 17 | **Polarisation-electric field loops of 0.5 mm-thick bulk PST sample 1 at different temperatures T (from 288 K to 313 K)**. The measurements were done using a standard Sawyer-Tower circuit at 20 Hz up to 18 kV cm^{-1} . The blue area is $W = \int E\mathrm{d}D$, which represents the electrical work per volume unit to charge PST at 299 K up to 18 kV cm^{-1}.

As shown in SI Fig. 12, PST is ferroelectric at 288 K and paraelectric at 313 K. Between 299 K and 305 K (SI Fig. 12) we observe double loops, which suggests antiferroelectricity. However, as already observed and explained by Shebanov [5] and then by Crossley [3], these double loops are evidences of a paraelectric to ferroelectric electric field-driven phase transition. Note that it was observed in 1953 in $BaTiO_3$, another electrocaloric material with a first order phase transition [13]. This phase transition is responsible for the high electrocaloric effect observed. Hence, Shebanov wrote that PST experiences a "field-induced first order phase transition manifested by the appearance of double dielectric hysteresis loops" that "provides a dominant contribution to the EC effect" [3].

Regarding the presence of double loops, the EC properties of lead zirconate (PZO), a true antiferroelectric material, can be mentioned and compared with PST. In PZO, antiferroelectricity infers a very peculiar electrocaloric effect. In [14], we observed double loops in PZO ceramics, somehow similar to the ones in PST. We showed though that by increasing temperature, PZO goes sequentially from antiferroelectric to ferroelectric and then to paraelectric. These successive phase transitions can be driven by an external electric field that gives rise to an outstanding negative electrocaloric effect when PZO goes from antiferroelectric to ferroelectric, a transition that has not been observed in PST.

Ferroelectric hysteresis infers losses. They have *de facto* been considered in our calculation of materials efficiency because we measured the current needed to charge PST. Our method is equivalent to considering not only the hysteresis losses but the entire work needed to fully charge the capacitor. More specifically, our experimental extraction is equivalent to using the lower branches of the P(E) loops to calculate the electrical work $W = \int E\mathrm{d}D$, as depicted by the blue shaded area of the *PE* loop at 299 K in Supplementary Fig. 17. Note that we used the term "*PE* loops" though these loops are "*DE* loops", *D* being the electrical displacement field. However, this is standard practise to consider *P* as equivalent to *D* in the case of high dielectric constant materials such as PST.

Supplementary Fig. 18 represents the electric work W_e extracted respectively from the direct method (same as Fig. 3c) and from the *PE* loop method for an electric field of 18 kV cm^{-1}. It shows that both methods give very similar results. This is no surprise as both involve current measurements, *P* being an integration of the current in PST while charging. Consequently, hysteresis losses are fully taken into account in our materials efficiency.

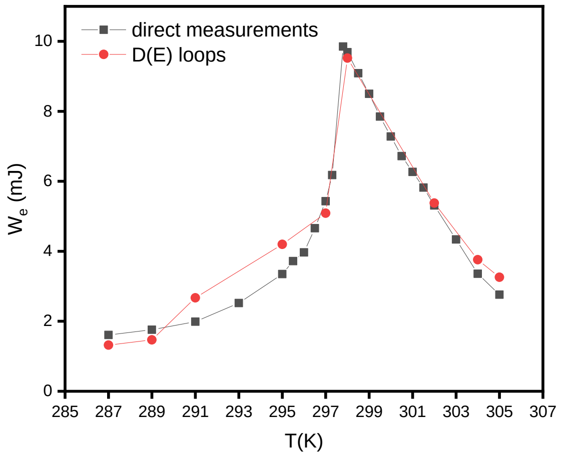

Supplementary Figure 18 | **Electrical work needed to charge PST up to 18 kV/cm from the direct method (charge at constant current) and from integrating *EdD* from a *DE* loop.**

Supplementary Note 13: Structure of PST bulk material

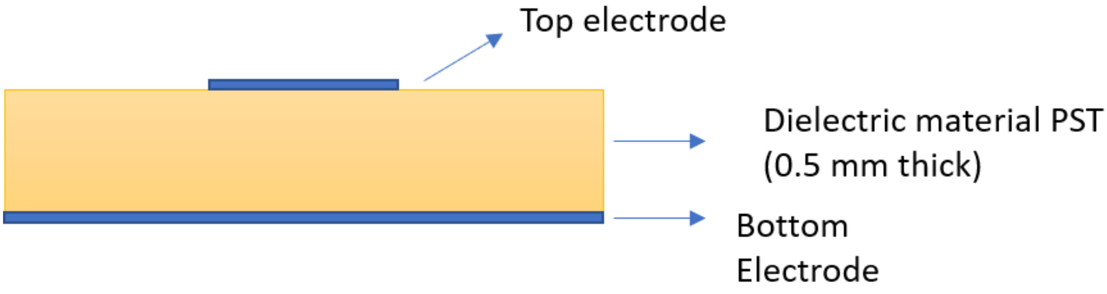

Supplementary Figure 19 | **Structure of PST bulk material.** The dielectric material PST is 0.5mm thick. The area of the top electrode is 0.480cm².

Supplementary Note 14: Potential Application of bulk PST

We show below how our PST could be used in a fluid-based regenerator without being affected by the asymmetry of ΔT_{adiab}, irreversibility or hysteresis losses. From ΔT_{adiab} measurements (Supplementary Fig. 20c) one can define a window of temperature where the regenerator could operate. At the maximum materials efficiency obtained at 11 kV cm^{-1}, we could simulate experimentally reproductible and reversible regenerators (Supplementary Fig. 20a and 20b). The regenerator would operate in a small temperature span at a lower heat exchanged (Supplementary Fig. 20d). The heat can be increased by increasing the applied electric field but the materials efficiency will decrease as the heat saturates while the electrical work increases (Supplementary Fig. 22).Furthermore, using the ΔT_{adiab} measurements in Supplementary Fig. 20c, we did some simulations of a fluid-based regenerator based on a similar model published in [15] operating at different starting temperatures around room temperature and without any kind of heat losses to the environment. We show that a temperature gradient can be reached in all cases after several cycles (Supplementary Fig. 21). This indicates that, despite an asymmetry in ΔT_{adiab} or hysteresis losses, bulk PST could be used to build different kinds of regenerator prototypes.

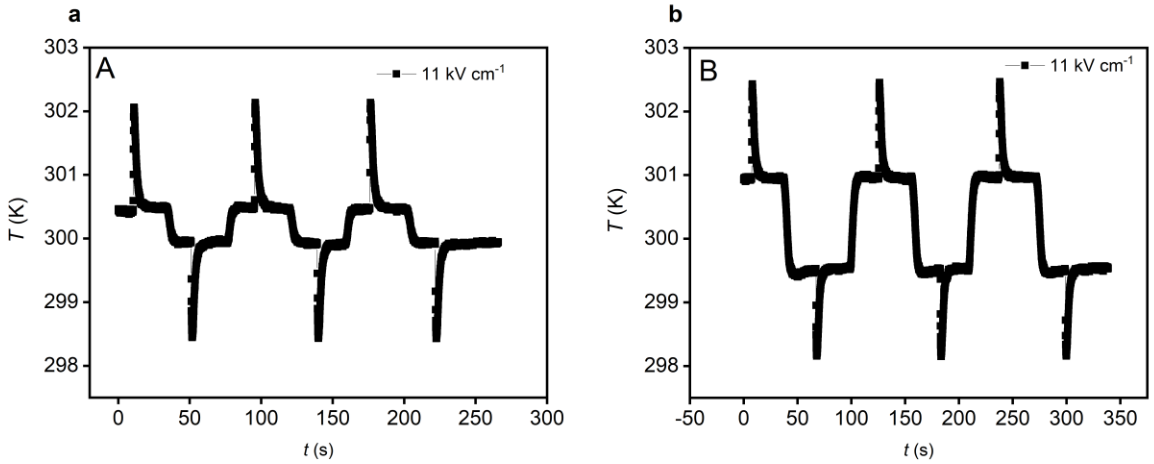

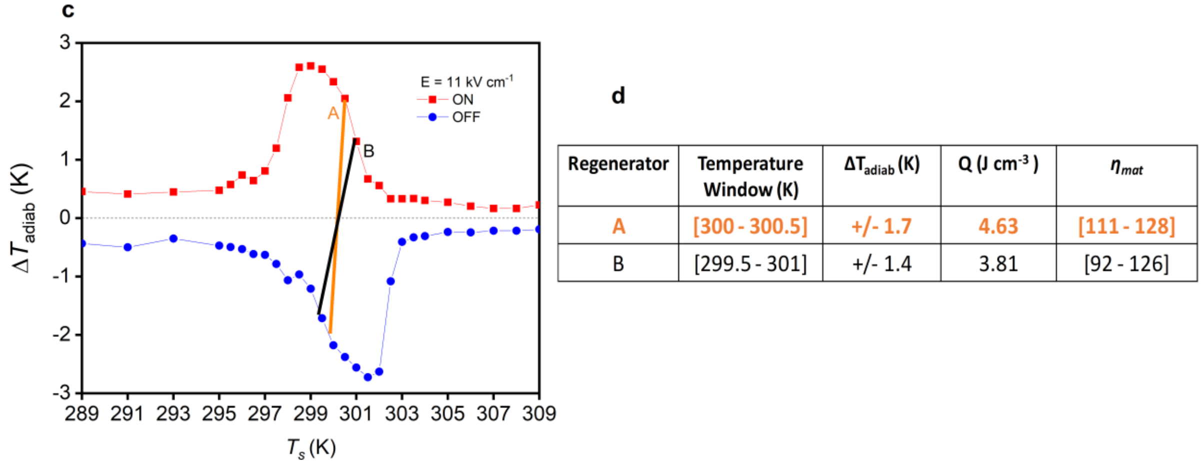

d

Regenerator	Temperature Window (K)	ΔT_{adiab} (K)	Q (J cm^{-3})	η_{mat}
A	[300 - 300.5]	+/- 1.7	4.63	[111 - 128]
B	[299.5 - 301]	+/- 1.4	3.81	[92 - 126]

Supplementary Figure 20| **Potential use of PST bulk in a regenerator.** Here we show two potential working points of PST bulk if it were integrated in a regenerator. The cycle of the latter has been mimicked with a temperature-controlled hot plate stage (Linkam). In **a** (resp. **b**), PST is first set at 300.5 K (resp. 301 K). The EC positive ΔT_{adiab} = 1.7 K (resp. 1.4 K) is then triggered by charging PST. Heat is exchanged and PST goes back to 300.5 K (resp. 301 K). If PST was in a regenerator, a fluid (for instance) would then be displaced and PST temperature would decrease. Here, we suppose that this temperature is 300 K (resp. 299.5 K). This value is chosen in order to obtain a symmetrical position in ΔT_{adiab} = f(T_s) displayed in c. This EC negative ΔT_{adiab} = -1.7 K (resp. -1.4 K) is then triggered by discharging PST and PST exchanges heat until it reaches 300 K (299.5 K) again. And the cycle carries on as it would in a proper regenerator. **a)** Regenerator A operating between 300-300.5 K, on a temperature window of 0.5 K. **b)** Regenerator B operating on a temperature window of 1.5 K. **c)** Adiabatic temperature change of PST bulk at 11 kV cm^{-1}. The orange and black lines represent respectively the regenerators A and B. **d)** the table shows for each regenerator, its temperature window, the heat exchanged and the corresponding measured materials efficiency.

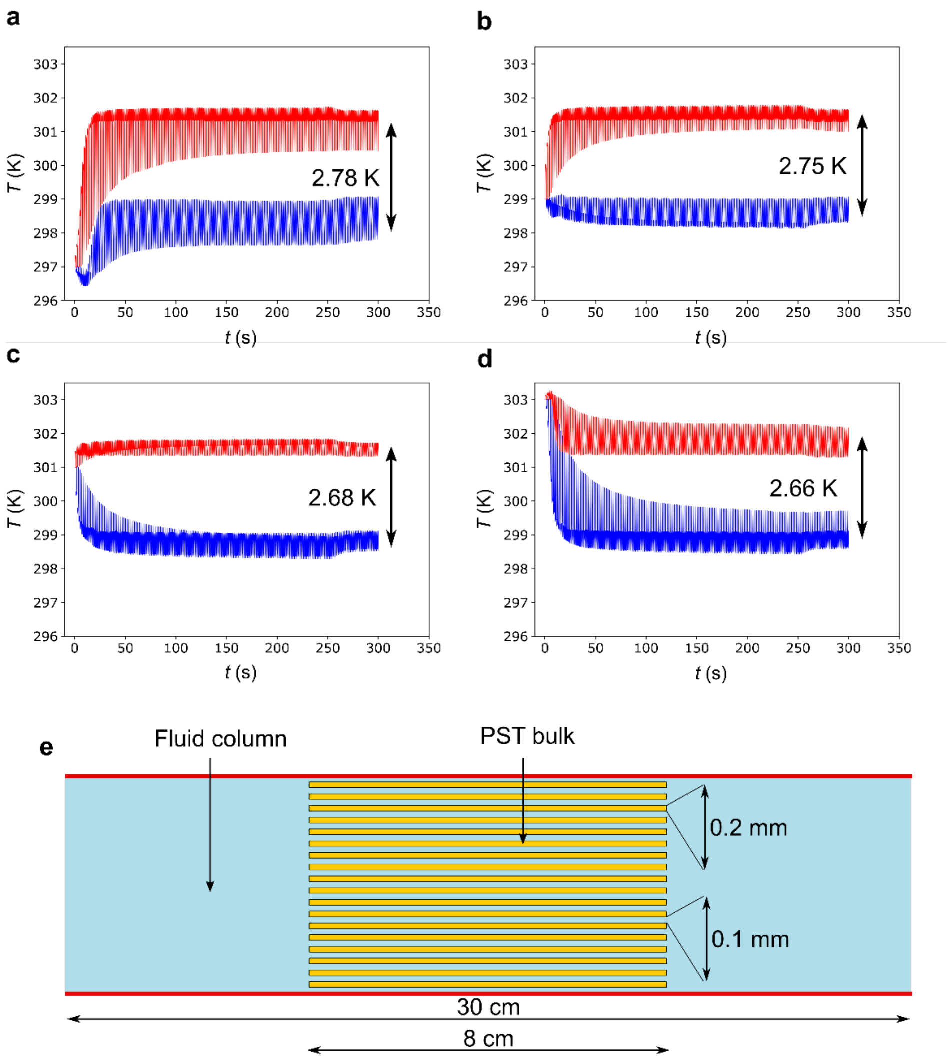

Supplementary Figure 21| **Regenerator modelling.** The time evolution of the hot side (red) and cold side (blue) of an active regenerator based on the EC effect from Supplementary Fig. 20c at starting temperatures (**a)** 297 K, **b)** 299 K, **c)** 301 K and **d)** 303 K. **e)** shows the dimensions of the regenerator simulated. The simulation consists of a finite element method (FEM) 2D representation of an active regenerator made with a single PST plate of 0.2 mm x 4 cm. No losses to the surroundings were considered. All the parameters of this model are detailed in [15].

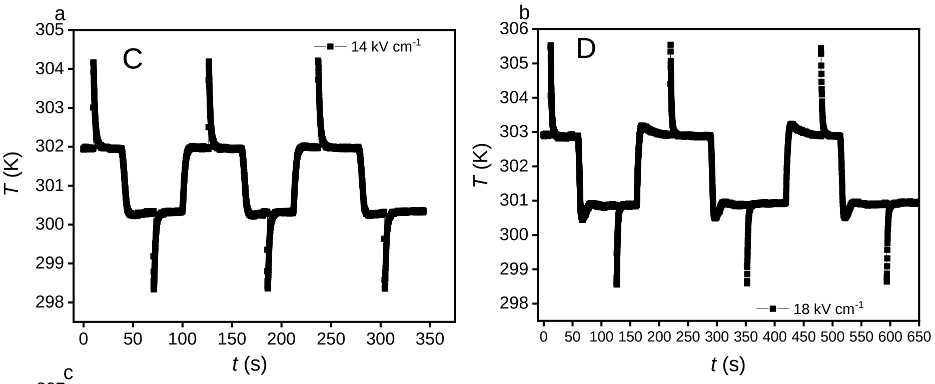

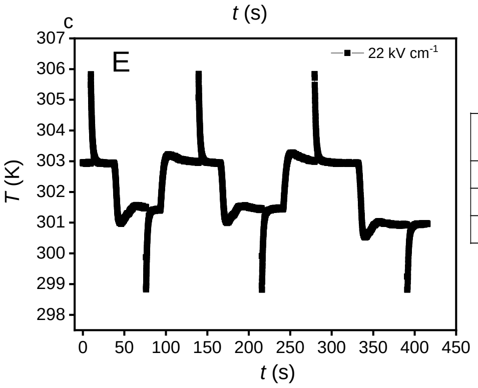

d

Regenerator	Temperature window (K)	ΔT_{adiab} (K)	Q (J cm^{-3})	η_{mat}
C	[300.5-302]	+/- 2.21	+/- 6.2	[62-75]
D	[301-303]	+/- 2.6	+/- 7	[31-41]
E	301.5-303	+/- 2.9	+/- 8	[19-24]

Supplementary Figure 22| **Potential regenerator for PST bulk at higher heat Q.** The heat exchanged can be increase by increasing the electric field but this will decrease the materials efficiency as shown in **d)**. **a)** regenerator C at the electric field of 14 kV cm^{-1} **b)** regenerator D at 18 kV cm^{-1} **c)** regenerator E at field of 22 kV cm^{-1} **d)** for each regenerator, the heat exchanged, materials efficiency and adiabatic temperature change.

Supplementary Note 15: Electrocaloric Exchangeable heat

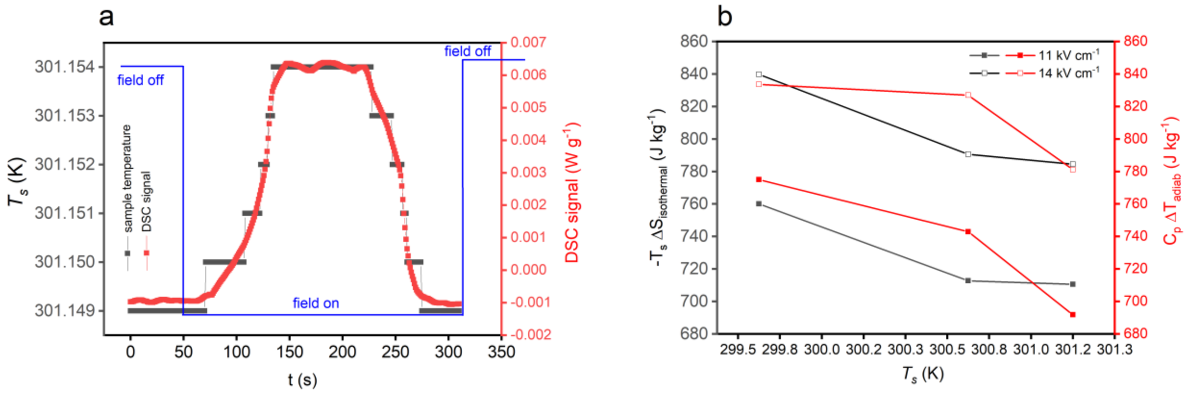

Supplementary Figure 23 | **Comparison between C_p ΔT_{adiab} and -T_s $\Delta S_{isothermal}$. a)** Isothermal application of an electric field of 14 kV cm^{-1} using DSC. It is applied very slowly (200 s) to maintain the temperature almost constant and measure a DSC signal. The integral under the DSC signal corresponds to the isothermal heat exchange -$T_s\Delta S_{isothermal}$. **b)** comparison of C_p ΔT_{adiab} to -T_s $\Delta S_{isothermal}$ at two electric field values (11 and 14 kV cm^{-1}). ΔT_{adiab} is measured with the IR camera in adiabatic conditions

and C_p is considered as constant (300 J kg^{-1} K^{-1}), which corresponds to the background value of C_p measurements (figure 1b). The very good match between C_p ΔT_{adiab} to -T_s $\Delta S_{isothermal}$ proves that it is legitimate to consider C_p ΔT_{adiab} as an excellent estimation of the heat exchanged in $\eta_{mat.}$

Supplementary References

[1] Shebanov, L., Birks, E. H. & Borman, K. X-ray studies of electrocaloric lead scandium tantalate ordered solid solutions. *Ferroelectrics* **90**, 165-172 (1989).

[2] Wang, H. and Schuize, W.A. Order-Discorder Phenomenon in Lead Scandium Tantalate. *J. Am. Ceram. Soc.* **73**, 1228 (1990).

[3] Crossley, S., Nair, B., Whatmore, R. W., Moya, X. & Mathur, N. D. Electrocaloric cooling cycles in lead scandium tantalate with true regeneration via field variation. *Phys. Rev. X* **9**, 041002 (2019).

[4] Shebanov, L. & Borman, K. On lead-scandium tantalate solid solutions with high electrocaloric effect. *Ferroelectrics* **127,** 143–148 (1992).

[5] Shebanov, L., Sternberg, A., Lawless, W. N. & Borman, K. Isomorphous ion substitutions and order–disorder phenomena in highly electrocaloric lead-scandium tantalate solid solutions. *Ferroelectrics* **184**, 239–242 (1996).

[6] Bjørk, R., Bahl, C.R.H. & Katter, M. Magnetocaloric properties of $LaFe_{13\_x\_y}$ Co_xSi_y and commercial grade Gd. *Journal of Magnetism and Magnetic Materials* ***322***, 3882-3888 (2010).

[7] Nielsen, K. K., Bahl, C. R. H. & Smith, A. Constraints on the adiabatic temperature change in magnetocaloric materials. *Physical Review B* **81**, 054423(1-5) (2010).

[8] Nair, B., Ph.D. thesis, University of Cambridge, 2020, available at https://www.repository.cam.ac.uk/handle/1810/312805

[9] Nair, B. *et al.* Large electrocaloric effects in oxide multilayer capacitors over a wide temperature range. *Nature* **575**, 468-472 (2019).

[10] Stern-Taulats, E., PhD Thesis, *Universitat de Barcelona* (2017)

[11] Stern-Taulats, E. et *al.*, *APL* **107**, 152409 (2015)

[12] Crossley, S., Mathur, N.D. & Moya, X. New developments in caloric materials for cooling applications. *AIP Advances* **5**, 061753 (2015).

[13] Merz, W.J., Double hysteresis loop of BaTiO3 at the Curie point. Phys. Rev. **91**, 513 (1953).

[14] Vales-Castro, P. et al, Origin of large negative electrocaloric effect in antiferroelectric $PbZrO_3$ Phys.Rev. B **103**, 054112 (2021).

[15] Torelló, A. *et al.* Giant temperature span in electrocaloric regenerator. *Science* **370**, 125-129 (2020).
